# The Cost-Effectiveness of an Advanced Hybrid Closed-Loop System Compared to Standard Management of Type 1 Diabetes in a Singapore Setting

**DOI:** 10.1089/dia.2023.0455

**Published:** 2024-04-30

**Authors:** Daphne Gardner, Mrinmayee Lakkad, Zhiyu Qiu, Yuta Inoue, Suresh Rama Chandran, Kael Wherry

**Affiliations:** ^1^ Department of Endocrinology, Singapore General Hospital, Singapore, Singapore.; ^2^ Medtronic Diabetes, Northridge, California, USA.; ^3^ Medtronic Singapore, Singapore, Singapore.

**Keywords:** Advanced hybrid closed-loop system, Cost-effectiveness, Cost of days off work, Type 1 diabetes, Singapore

## Abstract

**Background:**

Despite advances in technology, glycemic outcomes in people with type 1 diabetes (T1D) remain suboptimal. The MiniMed 780G (MM780G) advanced hybrid closed-loop (AHCL) system is the latest technology for T1D management with established safety and efficacy. This study explores the cost-effectiveness of MM780G AHCL compared against multiple daily injections (MDI) plus intermittently scanned continuous glucose monitor (isCGM).

**Methods:**

A cost-utility analysis was conducted, simulating lifetime outcomes for 1000 T1D individuals, with baseline hemoglobin A1c of 8.4%, using the IQVIA Core Diabetes Model (CDM) v9.5. A Singapore health care payer perspective was taken with 2023 costs applied. Treatment effects were taken from the ADAPT study and treatment-related events from a combination of sources. T1D complication costs were derived from local literature, and health state utilities and disutilities from published literature. Scenario analyses and probabilistic sensitivity analyses (PSAs) explored uncertainty. Cost-effectiveness was assessed based on willingness-to-pay (WTP) thresholds set to Singapore Dollars (SGD) 45,000 (United States Dollars [USD] 33,087) per quality-adjusted life year (QALY) and Singapore's gross domestic product (GDP) per capita of SGD 114,165 (USD 83,941) per QALY.

**Results:**

A switch from MDI plus isCGM to MM780G resulted in expected gains in life-years (+0.78) and QALYs (+1.45). Cost savings through reduction in T1D complications (SGD 25,465; USD 18,723) partially offset the higher treatment costs in the AHCL arm (+SGD 74,538; +USD 54,805), resulting in an estimated incremental cost-effectiveness ratio of SGD 33,797 (USD 24,850) per QALY gained. Findings were robust, with PSA outputs indicating 81% and 99% probabilities of cost-effectiveness at the stated WTP thresholds.

**Conclusion:**

MM780G is a cost-effective option for people with T1D managed in a Singapore setting.

## Introduction

Diabetes mellitus (DM), consisting of type 1 (T1D) and type 2 diabetes, comes with a significant global disease burden and is a growing problem in Singapore. Poorly controlled diabetes places a high burden on health systems, with health care expenditure and productivity losses expected to grow substantially over the next decade, along with increased incidence of diabetes complications.^1^

People with T1D have a significant burden of self-care due to the need for insulin replacement from diagnosis and high glucose variability.^2^ According to the SingHealth Diabetes Registry (SDR), the number of people being treated for T1D saw a 2.3-fold increase in less than 10 years, from 2013 to 2020, with the number of adult patients reaching 1300 in 2020 in SDR.^3^ The SDR gathers data from 193,000 people with diabetes across the SingHealth health cluster in Singapore. In 2016, a “War on Diabetes” policy was declared by Singapore's Ministry of Health (MOH), emphasizing diabetes as a priority condition.^4^ This policy aimed to reduce the growing burden of diabetes and was accompanied by value-based approaches to improve access to and address gaps in care. These included the prioritization of therapies and devices that have demonstrated improved outcomes.^5^

For people living with T1D, ideal glycemia is defined not just according to hemoglobin A1c (HbA1c) (which only describes mean glycemia) but also stable glycemia with minimal hypoglycemia (or time-below-range).^6^ This is encapsulated within the international consensus guidelines on time-in-range (TIR) using continuous glucose monitoring data, with >70% time spent in TIR of 3.9–10 mmol/L being considered an optimal target and <4% of the time spent below range (TBR) of 3.9 mmol/L. Self-management in T1D involves using real-time glucose data to make decisions about insulin dosing. These decisions consider factors such as carbohydrate intake and physical activity. In addition, they may also be influenced by factors like being sick or the menstrual cycle.^7^

Advancements in glucose testing platforms from point-of-care capillary blood glucose meters toward intermittently scanned continuous glucose monitor (isCGM) or real-time CGM now offer accurate, real-time, easily accessible glucose data, and have been demonstrated to improve outcomes.^8–10^ Similarly, insulin delivery systems have progressed from multiple daily injections (MDI) toward continuous subcutaneous insulin infusion (CSII), demonstrating improved glycemic control in those who continue to use them.^11–13^ However, with open-loop systems, where insulin delivery and glucose detection work independently, the burden of insulin dose titration still falls upon the person living with T1D and their health care professionals. Even when considering the various factors that influence insulin requirements, there remains much variability in daily insulin requirements in T1D, underpinning the challenges in sustaining stable and optimal mean glycemia.

Therefore, despite education in T1D self-management,^14,15^ advances in insulin pump technology, and rapid growth of CGM use, glycemic outcomes among people with T1D remain suboptimal. The US T1D Exchange registry (*n* = 25,529) showed that mean HbA1c did not improve over an 8-year period, with only 17% of adolescents and 21% of adults achieving the American Diabetes Association's (ADA) target HbA1c goal of <7%.^12^ The UK NHS National Diabetes Audit reported in July 2022 revealed that >65% of UK adults still had an HbA1c of >7.5%.^16^ In addition, a 2021 study involving Singaporean T1D individuals reported even less favorable findings, with suboptimal glycemic levels in 75% of young adults included in the study.^17^ Similarly, many individuals struggled to achieve recommended TIR targets of >70%.^9^

Hybrid closed-loop (HCL) technology integrates a CGM with an insulin pump and an algorithm to automatically adjust basal insulin in response to sensor glucose changes and predictively adjust insulin delivery to minimize hypoglycemia and hyperglycemia. Meal announcements with manual boluses for meals are still required. The effectiveness of these systems in targeting hyperglycemia, while preventing hypoglycemia, has been proven in both clinical trials and real-world evaluations.^18,19^ The latest iteration, the MiniMed 780G system (MM780G), is an advanced HCL (AHCL) system, which integrates an additional function of automated correctional boluses to further address hyperglycemia (>6.7 mmol/L) and may be delivered every 5 min. The safety and efficacy of MM780G have been demonstrated in a variety of clinical studies; perhaps more importantly, its continued use in real-world setting points toward user acceptability.^20–23^

In users already using isCGM plus MDI, yet not achieving HbA1c targets, the use of MM780G resulted in a substantial improvement in HbA1c (1.54%, standard deviation [SD]: 0.73) from a baseline of 9%.^19,24^ These improvements in glycemia were achieved without increased TBR. Indeed, in those with impaired awareness of hypoglycemia, despite using a form of diabetes technology (isCGM/CSII or sensor-augmented pump), the use of MM780G led to both improvement of hypoglycemia awareness and HbA1c levels.^25^ The benefit of automated insulin delivery systems notwithstanding, access to these systems remains limited in many parts of the world. This is particularly pertinent in countries where there are limited reimbursement policies for those living with diabetes, with all costs for devices borne by the person with T1D.

In Singapore, the Healthier SG movement aims to prioritize preventative health care to reduce the downstream burden from complications related to suboptimal management of noncommunicable diseases.^26^ Accordingly, it is important to consider the costs of adopting this technology versus the benefits over a protracted time trajectory. The cost-effectiveness of MM780G has been assessed in two European countries,^27,28^ but the health care structure of these settings and the tariffs applied in these analyses limit the ability to generalize these findings to other settings. This study explores the cost-effectiveness of MM780G for the management of T1D in a Singapore setting, which offers no reimbursement for CGM or AHCL systems, akin to neighboring countries in the region. Currently, available data are applied to a localized analysis setting.

## Methods

We conducted a cost-utility analysis, simulating lifetime outcomes for 1000 people with T1D using the IQVIA Core Diabetes Model v9.5 (CDM)^29–31^ to estimate the cost-effectiveness of MM780G compared to MDI plus isCGM in a Singapore setting.

### The economic model

The CDM T1D model consists of a series of Markov submodules that simulate the expected management pathway of people with T1D based on the likelihood of T1D complications (the model health states). Each health state is assigned an annual cost and a weighted utility to enable the estimation of an incremental cost-effectiveness ratio (ICER). Progression through the model (i.e., the T1D pathway) is defined according to the baseline characteristics of the cohort and the expected treatment impact of the modeled management strategies (based on extrapolation of the short-term impact on HbA1c).

Model outcomes are estimated for each management strategy (e.g., usual care vs. a novel intervention) and include projected lifetime direct treatment costs arising from diabetes-related complications and T1D management, indirect costs from lost workplace productivity, and health benefits. Health benefits are based on the estimated rates of T1D complications and the expected impact on both total life years (life expectancy) and quality-adjusted life years (QALYs). Cost-effectiveness is assessed based on a calculation of the ICER associated with a switch in management strategy (i.e., the difference in costs divided by the difference in QALYs).

### Perspective, time horizon, and discounting

The analysis was conducted from the Singapore health care payer perspective with 2023 costs applied.^32^ A lifetime time horizon was applied with an annual discount rate of 3% applied to both costs and outcomes, following Singapore's Agency for Care Effectiveness (ACE)'s technical guidance.^33^ Scenario analysis explored the inclusion of costs related to productivity losses from T1D morbidity and complications, alongside the baseline perspective of direct medical costs alone, as a proxy for a societal perspective.

### Patient population

The patient population comprised adults with T1D suitable for MM780G. Cohort characteristics including gender (76.0% female), baseline HbA1c (mean: 8.4%, SD: 1.84%), and mean age (32.2 years, SD: 15.9 years) were taken from the Singapore General Hospital (SGH) database, a main center for management of T1D in Singapore and the only center in Asia that is DAFNE accredited,^34^ a structured group education program for people with T1D, globally recognized as a gold standard of care.

### Intervention and comparator

MM780G was compared against MDI plus isCGM, a commonly used care method for people with T1D with a mean HbA1c of 8.4%.^35^

### Treatment effects and treatment-related events

Treatment effects were taken from the ADAPT trial^19^ and treatment-related events were estimated from a combination of sources.^19,27,36,37^ In MM780G, HbA1c was reduced by 1.54%, while in the MDI plus isCGM, the reduction was 0.2%.^19^ The baseline rates of severe hypoglycemia events (SHE) requiring another person's assistance, but not requiring medical assistance (SHE 1), SHE requiring medical assistance (SHE 2), and diabetic ketoacidosis (DKA) were estimated at 64.6, 13.0, and 2.93 per 100 patient-years for MDI plus isCGM, respectively, based on an average of reported values.^37–39^ We assumed a conservative 50% reduction^40^ in SHE and DKA with the use of AHCL versus MDI plus isCGM, noting that ADAPT recorded zero SHE and DKA events. Inputs were validated by the clinical authors based on local experience of T1D management (Table 1).

**Table 1.** Treatment Effects and Treatment-Related Events

Parameter	AHCL	MDI + isCGM	References
Change in HbA1c	−1.5 (0.73)%	−0.2 (0.80)%	^19^
Diabetes-related events (per 100 patient-years)^a^			
NSHE	970.84	1941.68	NICE T1D guidelines^68^
SHE 1	32.30	64.60	^38^
SHE 2	6.50	13.00	^37^
DKA	1.47	2.93	^39^

^a^ MM780 event rates assume a 50% reduction in SHE/DKA for AHCL.

AHCL, advanced hybrid closed loop; DKA, diabetic ketoacidosis; HbA1c, hemoglobin A1c; MDI, multiple daily injections; NICE, National Institute for Health and Care Excellence; NSHE, nonsevere hypoglycemia event; SHE 1, severe hypoglycemia event not requiring medical assistance; SHE 2, severe hypoglycemia event requiring medical assistance.

### Treatment costs

Treatment costs were estimated based on local clinical practice with the application of direct costs to the Singapore health care system.^33^ The annual cost of MDI plus isCGM was estimated at Singapore Dollars (SGD) 3829 (United States Dollars [USD] 2815), including insulin and insulin administration-related costs of SGD 1071 (USD 787). Insulin costs were estimated based on a total daily dose (TDD) of 0.71 per IU/kg (15,549 IU annual dose), based on a clinical expectation of T1D management in Singapore.^2^ Insulin use was assumed to be split 50/50 between rapid-acting [SGD 9.27 (USD 6.82) per 300 U] and long-acting insulin [SGD 7.99 (USD 5.87) per 300 U] formulations, with consumable costs based on an average of 5 CBG strips and lancets per week, based on local clinical practice.

The annual cost of AHCL was estimated at SGD 7566 (USD 5563), including insulin and insulin administration-related costs of SGD 1487 (USD 1093). AHCL costs were based on a 4-year use of a pump, 3 days use per reservoir, 7 days use per sensor, and no additional cost for transmitter kits. Insulin costs were estimated based on a 20% reduction in total insulin use with AHCL compared to MDI plus isCGM (a TDD of 0.57 IU/kg)^41–44^ and 100% use of rapid-acting insulin; consumable costs were based on 3-day use per infusion set, 5 CBG strips, and lancets per week. Priming-related insulin wastage was not considered in the analysis. A scenario analysis explored the impact of applying retail pharmacy costs for insulins and consumables (as opposed to public institution prices charged to patients).

### T1D complications costs

T1D complication costs (the model health state costs) were derived from local literature based on searches of the Tufts Cost-Effectiveness Analysis (CEA) Registry (www.cearegistry.org) (combining the complication terms with the country identifier Singapore) and the PubMed database (https://pubmed.ncbi.nlm.nih.gov) (using the search strategy T1D and Singapore). A core article was identified, which provided cost estimates for diabetes-related complications,^45^ with these inputs supplemented by data from more recent Singapore cost-effectiveness publications, where available.^46–49^ Scenario analysis considered the inclusion of indirect costs (limited to lost productivity) to approximate a societal perspective.

The cost of days off work (DOW) was estimated based on a mean monthly wage of SGD 5070 (USD 3728) for males in 2022 and SGD 4388 (USD 3226) for females in 2022.^50^ The DOW was conservatively approximated with inpatient length of stay for diabetes-related complications taken from published MOH data.^49^ These do not include DOW required for subsequent follow-up visits, home recovery, or rehabilitation, and hence underestimate the total DOW. Costs were inflated to 2023 SGD using the gross domestic product (GDP) implicit price deflator published by the International Monetary Fund.^32^ For November 2023, exchange rates for SGD and USD were set at SGD 1.36 to 1 USD,^51^ and additional currency conversions are provided in Supplementary Table S1. All cost inputs were validated by the clinical authors (Table 2).

**Table 2.** Type 1 Diabetes Complication Costs (Health State Costs)

Complication	Direct cost (2023 SGD*^a^*, USD*^b^*)		References	Total DOW*^c^*
	Year 1	Year 2+		
Myocardial infarction	27,090 (19,919)	1567 (1152)	^46^	4.1
Angina	3427 (2520)	2045 (1504)	^45^	3.4
Congestive Heart Failure	8519 (6264)	2527 (1858)	^45^	0^d^
Stroke	24,209 (17,801)	4208 (3094)	^47^	9.8
Stroke (death within 30 days)	21,254 (15,628)	NA	^45^	9.8
PVD	31,774 (23,363)	2541 (1868)	^45^	4.5
Hemodialysis	59,660 (43,868)	50,907 (37,432)	^48^	26.0
Peritoneal dialysis	45,157 (33,204)	38,418 (28,249)	^48^	0^d^
Renal transplant	90,064 (66,224)	18,085 (13,298)	^48^	11.3
NSHE	19 (14)	NA	^45^	0^d^
SHE 1	19 (14)	NA	^45^	0^d^
SHE 2	5253 (3863)	NA	^45^	0^d^
Ketoacidosis event	11,373 (8363)	NA	^49^	0^d^
Laser treatment	2375 (1746)	NA	^45^	0^d^
Cataract operation	5616 (4129)	227 (167)	^45^	1.4
Blindness	21,993 (16,171)	306 (225)	^45^	0^d^
Peripheral neuropathy	419 (308)	366 (269)	^45^	0^d^
Active ulcer	2541 (1868)	NA	^45^	11.1
Amputation	27,591 (20,288)	NA	^45^	21
Post-amputation (prosthesis)	3561 (2618)	NA	^45^	21

^a^ All costs are inflated to SGD 2023 values.

^b^ November 2023 exchange rates for SGD and USD were set at SGD 1.36 to 1 USD.^52^

^c^ Estimated DOW limited to mean days recorded for complication-related inpatient days.^51^

^d^ Where local data were not available, DOW entries were assumed to be zero following a very conservative approach.

DOW, days of work; NA, not applicable where the model did not require inputs; PVD, peripheral vascular disease; SGD, Singapore Dollars; USD, United States Dollars.

### Health state and treatment-related utilities

Health state utilities and the disutilities associated with adverse events were derived from the published literature, based on established practice for CDM analyses. In addition, following previous analyses,^22^ a utility benefit of 0.0432 was applied to the AHCL arm based on a reduction in fear of hypoglycemia (FoH). This was estimated based on ADAPT 6-month study findings that demonstrated a 5.4-point decrease in the hypoglycemic fear survey (HFS) for people using the AHCL therapy^22^ compared against people using MDI plus isCGM. ADAPT study 6-month findings were also used for the MDI plus isCGM arm that reported a 2.0-point decrease in the HFS.^22^ A 1-U increase in HFS score corresponded to a utility decrement of 0.016. An earlier study reported that a 1-U increase in HFS score corresponded to a utility decrement of 0.008.^52^

### Analysis outcomes

Model outcomes comprised lifetime cohort and per-person costs, outcomes, and QALYs plus estimations of ICERs. Cost-effectiveness was assessed against two willingness-to-pay (WTP) thresholds—one at SGD 45,000 (USD 33,087) per QALY, which is a threshold for medical technologies with positive subsidy recommendations in previous HTA evaluations,^50^ and the other is an inferred WTP threshold of SGD 114,165 (USD 83,941) per QALY gained since Singapore does not have an explicit WTP threshold (equivalent to one-time Singapore GDP per capita,^53^ a conservative marker of a GDP-based WTP threshold). This is also a commonly used WTP threshold in local cost-effectiveness studies.^46,54–56^

### Treatment of uncertainty

The IQVIA CDM projects lifetime outcomes based on short-term treatment effects and is subject to uncertainty. A series of sensitivity analyses was undertaken to explore the impact of uncertainty on model outcomes. Specifically, we assessed the impact of adjusting the AHCL treatment effect on HbA1c down to the lower limit of effect in the ADAPT study (−0.8%),^19^ adjusting the rates of expected SHE and DKA in the comparator arm to reflect locally expected rates of events (8.74 and 8.46 per 100 patient-years, respectively) with a conservative 50% reduction applied to estimate the comparative rates for AHCL, in line with our base case assumptions, modeling the impact of zero SHE or DKA events, in line with evidence from the ADAPT study and finally, assuming no insulin dose reduction in the AHCL cohort (i.e., applying a TDD insulin of 0.71 IU/kg across both management arms).

Alongside these analyses, we explored the impact of alternate cohort characteristics by changing the baseline HbA1c to simulate cohorts with higher baseline HbA1c levels (HbA1c of 9.0% and 9.5%). We ran an alternate combination scenario to simulate a cohort with a lower HbA1c baseline (8.0%) and an AHCL treatment effect (−1.0%). This scenario was based on an ad hoc analysis of the SGH data conducted by the clinical authors to represent the exploration of a plausible T1D subgroup. We also ran a scenario analysis based on ADAPT 12-month study findings in which we used the FoH utility of 0.0528 for AHCL and 0.0024 for MDI+isCGM.

Probabilistic sensitivity analysis (PSA) was conducted with input parameters varied between published limits (where data were available) and by ±20% for unit cost inputs (and where data were unavailable). PSA outputs were used to generate a scatterplot, plotting incremental cost and effectiveness pairs on the cost-effectiveness plane, and a cost-effectiveness acceptability curve (CEAC), plotting the probability of cost-effectiveness across a range of WTP thresholds, allowing a graphical representation of uncertainty.

## Results

### Base case analysis

In the base case analysis, a management switch for individuals with T1D from MDI plus isCGM to MM780G resulted in expected gains in life expectancy (+0.78 LYs) and quality of life (+1.45 QALYs), alongside an expected increase in costs (+SGD 49,073; USD 36,081), resulting in an estimated ICER of SGD 33,797 (USD 24,850) per QALY gained. Analysis outputs are reported in Table 3. Cost savings through reduction in T1D complications (SGD 25,465; USD 18,723) partially offset the higher treatment costs in the AHCL arm (+SGD 74,538; +USD 54,805).

**Table 3.** Summary of Cost-Effectiveness Analysis Comparing Advanced Hybrid Closed-Loop Versus Multiple Daily Injections Plus Intermittently Scanned Continuous Glucose Monitor

Outcomes (mean, 95% CI)	Intervention		Incremental difference
	AHCL	MDI plus isCGM	
Life years	18.77	17.99	0.78
QALYs	12.72	11.27	1.45
Direct costs (SGD, USD^a^)	267,343 (196,577)	218,270 (160,494)	49,073 (36,083)
Δ Cost/Δ QALY (direct costs only)	SGD 33,797 (USD 24,850)		

^a^ November 2023 exchange rates for SGD and USD were set at SGD 1.36 to 1 USD.^52^

CI, confidence interval; QALY, quality-adjusted life-year.

### Sensitivity and scenario analysis

In sensitivity analyses, ICERs ranged from SGD 27,548 (USD 20,255) per QALY (a higher baseline HbA1c with the same treatment effect as the base case) to SGD 46,909 (USD 34,490) per QALY (a conservative estimate using a lower limit of treatment effect from the ADAPT study), compared against a base case ICER of SGD 33,797 (USD 24,850) per QALY gained. Full results are reported in Table 4.

**Table 4.** Sensitivity and Scenario Analyses of Cost-Effectiveness Analysis Comparing Advanced Hybrid Closed-Loop Versus Multiple Daily Injections Plus Intermittently Scanned Continuous Glucose Monitor

Analysis	QALYs			Costs (SGD, USD*^a^*)			ICER based on combined costs, SGD per QALY gained	% Impact
	AHCL	MDI plus isCGM	Difference	AHCL	MDI plus isCGM	Difference		
Base-case	12.72	11.27	+1.45	267,343 (196,577)	218,270 (160,494)	+49,073 (36,083)	33,797 (24,850)	—
Sensitivity analyses								
SA 1	12.38	11.27	+1.11	270,690 (199,038)	218,270 (160,494)	+52,420 (38,544)	46,909 (34,492)	+39%
SA 2	12.47	10.98	+1.49	269,620 (198,252)	223,375 (164,248)	+46,245 (34,004)	30,940 (22,750)	−8%
SA 3	12.22	10.70	+1.52	271,935 (199,954)	229,974 (169,100)	+41,961 (30,854)	27,548 (20,256)	−18%
SA 4	12.74	12.31	+1.43	265,197 (194,999)	214,155 (157,468)	+51,042 (37,531)	35,686 (26,240)	+6%
SA 5	12.72	11.25	+1.47	273,277 (200,941)	229,476 (168,734)	+43,801 (32,207)	29,866 (21,960)	−12%
SA 6	12.79	11.27	+1.52	260,798 (191,765)	218,270 (160,494)	+42,528 (31,271)	27,906 (20,519)	−17%
SA 7	12.72	11.27	+1.45	269,299 (198,016)	218,270 (160,494)	+51,029 (37,522)	35,144 (25,841)	+4%
SA 8	12.90	11.02	+1.89	267,343 (196,577)	218,270 (160,494)	+49,073 (36,083)	26,010 (19,125)	−23%
Additional analyses								
Indirect costs (limited to lost productivity) analysis	12.72	11.27	+1.45	442,350 (325,260)	415,379 (305,428)	+26,971 (19,832)	18,575 (13,658)	−45%
Retail pricing	12.72	11.27	+1.45	277,642 (204,150)	243,739 (179,221)	+33,903 (24,929)	23,349 (17,169)	−31%
Subgroup analysis^b^	12.68	11.48	+1.20	267,919 (197,001)	214,621 (157,811)	+53,298 (39,190)	44,474 (32,702)	+32%

Base case: Baseline HbA1c: 8.4% AHCL treatment effect −1.5%, MDI/isCGM treatment effect −0.2%, DKA: 2.93/100 patient-years; MDI/isCGM SHE2: 13.00/100 patient-years; SA 1: AHCL treatment effect −0.8%; SA 2: Baseline HbA1c: 9.0%; SA 3: Baseline HbA1c: 9.5%; SA 4: MDI/isCGM SHE2: 8.74/100 patient-years; SA 5: MDI/isCGM DKA: 8.46/100 patient-years; SA 6: AHCL SHE2 set to zero patient-years; SA 7: AHCL no reduction in insulin; SA 8: FoH utility from 12-month data 0.0528 for AHCL and 0.0024 for MDI+isCGM.

^a^ November 2023 exchange rates for SGD and USD were set at SGD 1.36 to 1 USD.^52^

^b^ HbA1c baseline (8.0%) and AHCL treatment effect (−1.0%).

FoH, fear of hypoglycemia; ICER, incremental cost-effectiveness ratio.

When considering those scenarios that explored the impact of alternate baseline levels of HbA1c or AHCL treatment effect, ICERs ranged between the lower limit of SGD 27,548 (USD 20,255) (baseline HbA1c of 9.5%) and an upper limit of SGD 46,909 (USD 34,490). An approximation of a conservative subgroup where individuals had a lower baseline HbA1c of 8.0% at the initiation of the AHCL system with lower treatment effect (HbA1c −1.0%) led to the ICER at SGD 44,474 (USD 32,700) per QALY gained.

Alternate assumptions of SHE2 and DKA event rates resulted in ICERs ranging from SGD 27,906 (USD 20,518) (AHCL SHE2: zero patient-years) to SGD 35,686 (USD 26,238) (MDI/isCGM SHE2: 8.74/100 patient-years and AHCL SHE2: 4.37/100 patient-years).

When the costs of DOW due to T1D morbidity and complications were included in the analysis (to approximate a societal perspective), the cost difference between arms was reduced from +SGD 49,073 to +SGD 26,971 (+USD 36,081 to +USD 19,831) and the ICER re-estimated at SGD 18,575 (USD 13,657) per QALY gained. Using the FoH utilities from the combined data source of 12-month observation period led to the ICER at SGD 26,010 (USD 19,124).

### PSA outputs are reported in figure legends

**FIG. 1.**
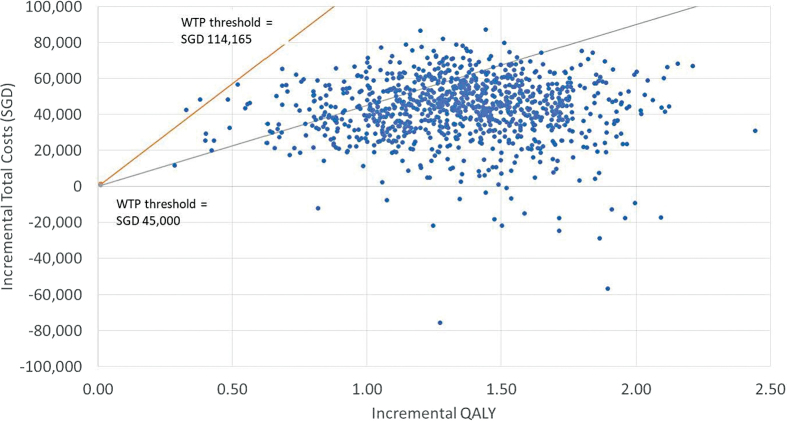
Probabilistic sensitivity analysis of CEA comparing AHCL versus MDI plus isCGM at WTP of SGD 45,000 per QALY (gray line) and SGD 114,165 per QALY (orange line) gained. AHCL, advanced hybrid closed loop; CEA, cost-effectiveness analysis; MDI, multiple daily injections; QALY, quality-adjusted life year; SGD, Singapore Dollars; WTP, willingness-to-pay. Color images are available online.

**FIG. 2.**
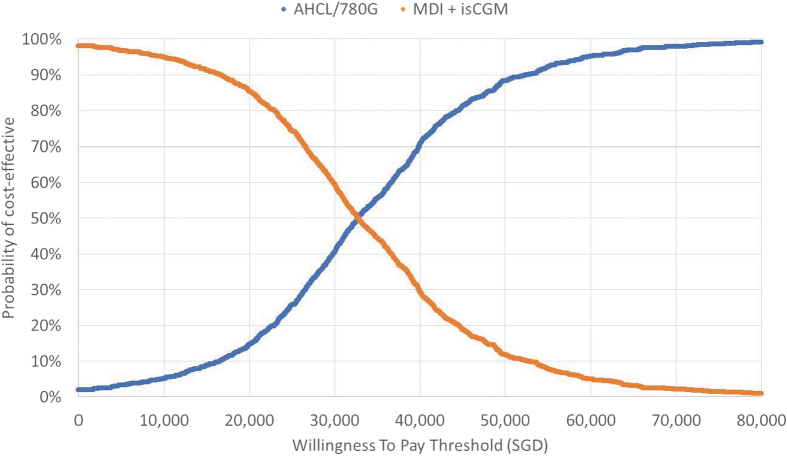
Cost-effectiveness acceptability curve comparing AHCL versus MDI plus isCGM. Cost-effectiveness was achieved at SGD 32,740/QALY (USD 24,072/QALY), where the lines cross each other. Where the line crosses for each therapy/system, is where the cost-effectiveness of one system (in this case the 780G) surpasses the comparator (MDI+isCGM). CEAC, cost-effectiveness acceptability curve; USD, United States Dollars. Color images are available online.

The PSA results showed an average QALY gain of 1.35 and a cost increase of SGD 42,779 (USD 31,454), close to the base case results (Figs. 1 and 2). The scatter plot and CEAC indicated that in the substantial majority (81% and 99%, respectively, at the WTP thresholds of SGD 45,000 (USD 33,087) per QALY and SGD 114,165 (USD 83,941) per QALY gained) of simulations, AHCL would be considered cost-effective. The CEAC indicated that AHCL would already be considered a cost-effective treatment when the threshold was above SGD 32,740/QALY (USD 24,072/QALY).

## Discussion

Our analysis found that in people in Singapore living with T1D, who are not achieving glycemic targets with isCGM and MDI, the use of the MiniMed 780G system was projected to lead to an incremental gain in QALY expectancy and quality of life. The reductions in the occurrence and cost of T1D complications partially offset the additional management costs of AHCL compared to MDI and isCGM, resulting in an estimated ICER of SGD 33,797 (USD 24,850) per QALY gained. Model outcomes were robust to exploration of uncertainty and AHCL remained a cost-effective management choice when inputs were altered within plausible limits. Based on the two inferred thresholds of WTP, and under current model assumptions, AHCL is a cost-effective management option for similar cohorts of T1D individuals managed in a Singapore setting.

Extensive sensitivity analyses were conducted. Model outcomes were most sensitive to the exploration of a lower treatment effect, which is expected.^24^ The difference between HbA1c improvements seen in the ADAPT study compared to other studies reflects the fact that the participants in ADAPT were specifically selected to reflect a cohort on MDI with high HbA1c, despite isCGM use, mapping well to the demographics of the SGH cohort from which baseline characteristics were modeled.^52,57^ As reported by Choudhary et al.,^19,24^ these patients represent an underserved section of the T1D population with a high capacity to benefit from improved outcomes with advanced technology. In assuming a lower baseline HbA1c of 8.0% with a lower treatment effect of −1.0%, the ICER remains comfortably under WTP thresholds.

The baseline assumptions for SHE and DKA for the MDI plus isCGM were estimated using data from large observational studies with a long follow-up duration of 12 months.^38,39^ Sensitivity analysis found that outcomes were not sensitive to alternate estimates of SHE and DKA rates obtained from the SDR database, which generated ICERs of SGD 35,686 (USD 26,238) per QALY and SGD 29,886 (USD 21,974) per QALY, respectively. An exploration of lower SHE2 event rate in the AHCL arm resulted in an ICER of SGD 27,906 (USD 20,518) per QALY gained. Future evidence generation could focus on a prospective assessment of rates of SHE and DKA in a Singapore setting over longer periods to better reflect a lifetime perspective.

The inclusion of indirect costs (limited to the costs of DOW due to T1D complications) resulted in an estimated ICER of SGD 18,575 (USD 13,657) per QALY gained. While this metric is not explicitly included in current reimbursement decisions in Singapore, recent moves toward the inclusion of patient perspectives and an emphasis on opportunities for involvement of people with T1D in ACE's decision making^58^ suggest that metrics such as DOW may start to play an increasing role in reflecting values that matter to people beyond the health care perspective.

It should also be noted that DOW estimation herein was limited to just inpatient episodes^40^ and is hence a conservative estimate, warranting additional exploration. The Singapore health care financing system is anchored on the dual philosophies of individual responsibility and affordable health care for all,^59^ and reductions in DOW are likely to benefit not only the individual but also society as a whole through enhancement of overall population-level productivity. The sensitivity analysis using 12-month data from ADAPT on FOH utility decrement also resulted in an ICER of SGD 26,010 (USD 19,124). This metric was within the implied thresholds of WTP and was robust to changes.

Costs of insulin treatment clearly influenced model outcomes. The base case included the insulin cost, which is subject to substantial group procurement discounts and is categorized under the Singapore standard drug list, and therefore eligible for subsidy and reimbursement. In those who are not eligible for subsidized health care within the Singapore setting, the retail costs of insulin are substantially higher by approximately two to five times. In this scenario, the differing cost in insulin pricing alone resulted in a lowering of the ICER to SGD 23,349 (USD 17,168) per QALY gained. This adds a further consideration for payers when evaluating the cost-effectiveness of AHCL versus MDI and isCGM therapy.

Previous analyses assessing the cost-effectiveness of AHCL (specifically, MM780G) are not directly comparable to this analysis as we specifically focused our analysis on people with high HbA1c. However, despite cohort differences, our findings are similar; a study conducted in Greece reported a direct cost ICER of EUR 29,869 (∼USD 31,028) based on a treatment effect of −0.8% HbA1c,^27^ while a study conducted in Sweden reported direct and indirect ICERS of SEK 430,663 and SEK 373,700, respectively (∼USD 40,822 and USD 35,425), based on a treatment effect of −0.5% HbA1c.^28^

In both cases, the authors concluded that AHCL was a cost-effective alternative to MDI plus isCGM^27^ or CSII.^28^ Evidence of cost-effectiveness is a key driver of national-level decision making around health care funding, and reimbursement of AHCL is currently expanding across European markets as the evidence base for use increases.^60^ For example, in June 2023, The National Institute for Health and Care Excellence (NICE) in United Kingdom recommended the use of HCL systems for T1D.^61^ In Singapore and neighboring countries, where the costs of CGM and AHCL systems are currently borne entirely by the person with T1D, due consideration could be given to increase access to these systems through reimbursement schemes, especially when the inclusion of indirect costs of complications is factored in.

There are some limitations to this analysis. Those which relate specifically to the use of long-term modeling in diabetes have been extensively addressed elsewhere,^62,63^ and will not be addressed further here. The baseline estimates for the rates of SHE and DKA were based on disparate , which were assessed to be the best available evidence derived from large-scale studies with long follow-up periods. We have, however, applied a conservative approach to the relative benefit of AHCL and explored the impact of our baseline assumptions in sensitivity analysis.

We also note potential limitations of the ADAPT dataset concerning its generalizability to a Singapore setting. The ADAPT study was conducted across a range of European settings and did not include a significant proportion of Asian patients.^18^ However, the study represented a robust head-to-head comparison of AHCL and MDI plus isCGM and represents the best available data for this patient population at the time of publication of this analysis. While future research could usefully generate data specific to regionally relevant populations, the use of ADAPT data, in combination with local costs, has allowed us to explore a robust and localized assessment of the cost-effectiveness of AHCL in a Singapore setting.

This study has attempted to make the most of available head-to-head data within the framework of an established and fully validated economic model and using, where possible, local input to tailor the model inputs to a Singapore T1D management setting. It is, however, useful to remind ourselves and emphasize the limitations of a CEA approach in capturing other “off-model” benefits of such AHCL systems, including the impact on self-care burden, the simplification of management complexity through the use of technology, user preferences for ease of management, and patient and carer anxiety associated with the potential for subsequent hypoglycemia events (as evidenced by improved hypoglycemia survey scores).^19,20,64–67^

The MM780G system addresses important unmet needs in T1D management, and the provision of a compelling economic argument for use is key to payer acceptance. Despite the limitations and caveats to the use of CEA, we provide here an analysis based on the iterative and collaborative involvement of local clinicians, using a robust and well-validated model, and providing strong support for the positive positioning of MM780G in the T1D management pathway in Singapore.

## Conclusion

This is the first CEA of the MM780 AHCL system in Asia, which provides valuable insights on the system's clinical and economic values to inform health care resource allocation and technology adoption. The analysis projected the expected lifetime costs and benefits of alternate management strategies for a cohort of people with T1D, not currently achieving target glycemia on MDI plus isCGM, and estimated an ICER of SGD 33,797 (USD 24,850) per QALY gained. From this, we can conclude that MM780G is a cost-effective option for people with T1D, suboptimally managed in a Singapore setting. Future research collating and reviewing locally generated data would further support robust policy decisions in this area.

## Supplementary Material

Supplemental data

## References

[1] International Diabetes Federation (IDF) Diabetes Atlas. Singapore Diabetes Report 2000—2045. n.d. Available from: https://www.diabetesatlas.org/data/ [Last accessed: March 31, 2023].

[2] Rama Chandran S, Tay WL, Lye WK, et al. Beyond HbA1c: Comparing glycemic variability and glycemic indices in predicting hypoglycemia in type 1 and type 2 diabetes. Diabetes Technol Ther 2018;20(5):353–362; doi: 10.1089/dia.2017.0388 29688755

[3] Tan JK, Salim NNM, Lim GH, et al. Trends in diabetes-related complications in Singapore, 2013–2020: A registry-based study. PLoS ONE 2022;17(10):e0275920; doi: 10.1371/journal.pone.0275920 36219616 PMC9553054

[4] Ministry of Health (MOH). MOH | News Highlights. n.d. Available from: https://www.moh.gov.sg/news-highlights/details/speech-by-minister-for-health-mr-gan-kim-yong-at-the-moh-committee-of-supply-debate-2016 [Last accessed: March 31, 2023].

[5] Nally LM, Sherr JL, Van Name MA, et al. Pharmacologic treatment options for type 1 diabetes: What's new? Expert Rev Clin Pharmacol 2019;12(5):471–479; doi: 10.1080/17512433.2019.1597705 30892094 PMC6488361

[6] Battelino T, Danne T, Bergenstal RM, et al. Clinical targets for continuous glucose monitoring data interpretation: Recommendations from the International Consensus on Time in Range. Diabetes Care 2019;42(8):1593–1603; doi: 10.2337/dci19-0028 31177185 PMC6973648

[7] Diaz C JL, Fabris C, Breton MD, et al. Insulin replacement across the menstrual cycle in women with type 1 diabetes: An in silico assessment of the Need for Ad Hoc Technology. Diabetes Technol Ther 2022;24(11):832–841; doi: 10.1089/dia.2022.0154 35714349

[8] Leelarathna L, Evans ML, Neupane S, et al. Intermittently scanned continuous glucose monitoring for type 1 diabetes. N Engl J Med 2022;387(16):1477–1487; doi: 10.1056/NEJMoa2205650 36198143

[9] Beck RW, Riddlesworth T, Ruedy K, et al. Effect of continuous glucose monitoring on glycemic control in adults with type 1 diabetes using insulin injections: The DIAMOND Randomized Clinical Trial. JAMA 2017;317(4):371–378; doi: 10.1001/jama.2016.19975 28118453

[10] Lind M, Polonsky W, Hirsch IB, et al. Continuous glucose monitoring vs conventional therapy for glycemic control in adults with type 1 diabetes treated with multiple daily insulin injections: The GOLD Randomized Clinical Trial. JAMA 2017;317(4):379–387; doi: 10.1001/jama.2016.19976 28118454

[11] Pickup JC, Reznik Y, Sutton AJ. Glycemic control during continuous subcutaneous insulin infusion versus multiple daily insulin injections in type 2 diabetes: Individual patient data meta-analysis and meta-regression of randomized controlled trials. Diabetes Care 2017;40(5):715–722; doi: 10.2337/dc16-2201 28428322

[12] Foster NC, Beck RW, Miller KM, et al. State of type 1 diabetes management and outcomes from the T1D Exchange in 2016–2018. Diabetes Technol Ther 2019;21(2):66–72; doi: 10.1089/dia.2018.0384 30657336 PMC7061293

[13] Nørgaard K, Scaramuzza A, Bratina N, et al. Routine sensor-augmented pump therapy in type 1 diabetes: The INTERPRET study. Diabetes Technol Ther 2013;15(4):273–280; doi: 10.1089/dia.2012.0288 23438304 PMC3696941

[14] SingHealth. Type 1 Diabetes Diet: Match Insulin Dosage with Carbohydrate Intake—HealthXchange. n.d. Available from: https://www.healthxchange.sg:443/diabetes/living-well-diabetes/type-one-diabetes-diet-insulin-carbohydrate [Last accessed: July 26, 2023].

[15] National Health Service (NHS). Dose Adjustment for Normal Eating (DAFNE). n.d. Available from: https://dafne.nhs.uk/ [Last accessed: July 26, 2023].

[16] Diabetes UK. National Diabetes Audit (NDA) Reports. n.d. Available from: https://www.diabetes.org.uk/professionals/resources/national-diabetes-audit/nda-reports [Last accessed: July 26, 2023].

[17] Zhu L, Chandran SR, Tan WB, et al. Persistent anxiety is associated with higher glycemia post-transition to adult services in Asian young adults with diabetes. Diabetes Metab J 2020;45(1):67–76; doi: 10.4093/dmj.2019.0226 32602276 PMC7850875

[18] Arrieta A, Battelino T, Scaramuzza AE, et al. Comparison of MiniMed 780G system performance in users aged younger and older than 15 years: Evidence from 12 870 real-world users. Diabetes Obes Metab 2022;24(7):1370–1379; doi: 10.1111/dom.14714 35403792 PMC9545031

[19] Choudhary P, Kolassa R, Keuthage W, et al. Advanced hybrid closed loop therapy versus conventional treatment in adults with type 1 diabetes (ADAPT): A randomised controlled study. Lancet Diabetes Endocrinol 2022;10(10):720–731; doi: 10.1016/S2213-8587(22)00212-1 36058207

[20] Carlson AL, Sherr JL, Shulman DI, et al. Safety and glycemic outcomes during the MiniMed™ Advanced Hybrid Closed-Loop System pivotal trial in adolescents and adults with type 1 diabetes. Diabetes Technol Ther 2022;24(3):178–189; doi: 10.1089/dia.2021.0319 34694909 PMC8971997

[21] Collyns OJ, Meier RA, Betts ZL, et al. Improved glycemic outcomes with Medtronic MiniMed Advanced Hybrid Closed-Loop Delivery: Results from a randomized crossover trial comparing automated insulin delivery with predictive low glucose suspend in people with type 1 diabetes. Diabetes Care 2021;44(4):969–975; doi: 10.2337/dc20-2250 33579715

[22] Bergenstal RM, Nimri R, Beck RW, et al. A comparison of two hybrid closed-loop systems in adolescents and young adults with type 1 diabetes (FLAIR): A multicentre, randomised, crossover trial. Lancet 2021;397(10270):208–219; doi: 10.1016/S0140-6736(20)32514-9 33453783 PMC9194961

[23] Silva JD, Lepore G, Battelino T, et al. Real-world performance of the MiniMed^TM^ 780G System: First report of outcomes from 4120 users. Diabetes Technol Ther 2022;24(2):113–119; doi: 10.1089/dia.2021.0203 34524003 PMC8817690

[24] de Portu S, Vorrink L, Re R, et al. Randomised controlled trial of Advanced Hybrid Closed Loop in an adult population with type 1 diabetes (ADAPT): Study protocol and rationale. BMJ Open 2022;12(2):e050635; doi: 10.1136/bmjopen-2021-050635 PMC8811581 35110310

[25] Nattero-Chávez L, Pascual EL, Calle EDL, et al. Switching to an advanced hybrid closed-loop system in real-world practice improves hypoglycemia awareness and metabolic control in adults with type 1 diabetes, particularly in those with impaired perception of hypoglycemia symptoms. Diabetes Res Clin Pract 2023;199:110627; doi: 10.1016/j.diabres.2023.110627 36940793

[26] Ministry of Health (MOH). What Is Healthier SG? n.d. Available from: https://www.healthiersg.gov.sg/about/what-is-healthier-sg/ [Last accessed: July 26, 2023].

[27] Lambadiari V, Ozdemir Saltik AZ, de Portu S, et al. Cost-effectiveness analysis of an Advanced Hybrid Closed-Loop Insulin Delivery System in people with type 1 diabetes in Greece. Diabetes Technol Ther 2022;24(5):316–323; doi: 10.1089/dia.2021.0443 34962140

[28] Jendle J, Buompensiere MI, Holm AL, et al. The cost-effectiveness of an Advanced Hybrid Closed-Loop System in people with type 1 diabetes: A Health Economic Analysis in Sweden. Diabetes Ther Res Treat Educ Diabetes Relat Disord 2021;12(11):2977–2991; doi: 10.1007/s13300-021-01157-0 PMC8519965 34596879

[29] Palmer AJ, Roze S, Valentine WJ, et al. The CORE Diabetes Model: Projecting long-term clinical outcomes, costs and cost-effectiveness of interventions in diabetes mellitus (types 1 and 2) to support clinical and reimbursement decision-making. Curr Med Res Opin 2004;20 Suppl 1:S5–S26; doi: 10.1185/030079904X1980 15324513

[30] Palmer AJ, Roze S, Valentine WJ, et al. Validation of the CORE Diabetes Model against epidemiological and clinical studies. Curr Med Res Opin 2004;20 Suppl 1:S27–S40; doi: 10.1185/030079904X2006 15324514

[31] McEwan P, Foos V, Palmer JL, et al. Validation of the IMS CORE Diabetes Model. Value Health 2014;17(6):714–724; doi: 10.1016/j.jval.2014.07.007 25236995

[32] Turner HC, Lauer JA, Tran BX, et al. Adjusting for inflation and currency changes within health economic studies. Value Health 2019;22(9):1026–1032; doi: 10.1016/j.jval.2019.03.021 31511179

[33] Agency for Care Effectiveness. Medical Technologies Evaluation Methods and Process Guide. 2022. n.d. Available from: https://www.ace-hta.gov.sg/resources/process-methods [Last accessed: March 31, 2023].

[34] Singapore General Hospital (SGH). Dose Adjustment for Normal Eating (DAFNE). n.d. Available from: https://www.sgh.com.sg:443/patient-care/specialties-services/Diabetes-and-Metabolism-Centre/Pages/DAFNE.aspx [Last accessed: March 31, 2023].

[35] Authors/Task Force Members, Rydén L, Grant PJ, et al. ESC Guidelines on diabetes, pre-diabetes, and cardiovascular diseases developed in collaboration with the EASD: The Task Force on diabetes, pre-diabetes, and cardiovascular diseases of the European Society of Cardiology (ESC) and developed in collaboration with the European Association for the Study of Diabetes (EASD). Eur Heart J 2013;34(39):3035–3087; doi: 10.1093/eurheartj/eht108 23996285

[36] Charleer S, De Block C, Nobels F, et al. Sustained impact of real-time continuous glucose monitoring in adults with type 1 diabetes on insulin pump therapy: Results after the 24-month RESCUE Study. Diabetes Care 2020;43(12):3016–3023; doi: 10.2337/dc20-1531 33067260

[37] Heinemann L, Freckmann G, Ehrmann D, et al. Real-time continuous glucose monitoring in adults with type 1 diabetes and impaired hypoglycaemia awareness or severe hypoglycaemia treated with multiple daily insulin injections (HypoDE): A multicentre, randomised controlled trial. Lancet 2018;391(10128):1367–1377; doi: 10.1016/S0140-6736(18)30297-6 29459019

[38] Charleer S, De Block C, Van Huffel L, et al. Quality of life and glucose control after 1 year of nationwide reimbursement of intermittently scanned continuous glucose monitoring in adults living with type 1 diabetes (FUTURE): A Prospective Observational Real-World Cohort Study. Diabetes Care 2020;43(2):389–397; doi: 10.2337/dc19-1610 31843948

[39] Roussel R, Riveline J-P, Vicaut E, et al. Important drop in rate of acute diabetes complications in people with type 1 or type 2 diabetes after initiation of flash glucose monitoring in France: The RELIEF Study. Diabetes Care 2021;44(6):1368–1376; doi: 10.2337/dc20-1690 33879536 PMC8247513

[40] Pease A, Callander E, Zomer E, et al. The cost of control: Cost-effectiveness analysis of hybrid closed-loop therapy in youth. Diabetes Care 2022;45(9):1971–1980; doi: 10.2337/dc21-2019 35775453

[41] Paldus B, Lee MH, O'Neal DN. Insulin pumps in general practice. Aust Prescr 2018;41(6):186–190; doi: 10.18773/austprescr.2018.056 30670886 PMC6299172

[42] Hoogma RPLM, Hammond PJ, Gomis R, et al. Comparison of the effects of continuous subcutaneous insulin infusion (CSII) and NPH-based multiple daily insulin injections (MDI) on glycaemic control and quality of life: Results of the 5-Nations Trial. Diabet Med 2006;23(2):141–147; doi: 10.1111/j.1464-5491.2005.01738.x 16433711

[43] Chico A, Tundidor D, Jordana L, et al. Changes in insulin requirements from the onset of continuous subcutaneous insulin infusion (CSII) until optimization of glycemic control. J Diabetes Sci Technol 2014;8(2):371–377; doi: 10.1177/1932296813520205 24876590 PMC4455399

[44] American Diabetes Association (ADA). Transitioning Patients with Type 1 Diabetes from Multiple Daily Injections (MDI) to Continuous Subcutaneous Insulin Infusion (CSII) | American Diabetes Association. n.d. Available from: https://professional.diabetes.org/abstract/transitioning-patients-type-1-diabetes-multiple-daily-injections-mdi-continuous [Last accessed: July 27, 2023].

[45] Goh SY, Tan SC, Lim LC, et al. Cost-effectiveness of switching from biphasic human insulin (BHI) to biphasic insulin aspart 30 (BIAsp-30) in type 2 diabetes patients with suboptimal glycaemic control in Singapore. J Diabetol 2015;6(1):2.

[46] Lin L, Teng M, Zhao YJ, et al. Long-term cost-effectiveness of statin treatment for primary prevention of cardiovascular disease in the elderly. Cardiovasc Drugs Ther 2015;29(2):187–197; doi: 10.1007/s10557-015-6584-7 25860556

[47] See-Toh RS-E, Wong XY, Mahboobani KSKH, et al. Cost-effectiveness of transcatheter aortic valve implantation in patients with severe symptomatic aortic stenosis of intermediate surgical risk in Singapore. BMC Health Serv Res 2022;22(1):994; doi: 10.1186/s12913-022-08369-5 35927703 PMC9354430

[48] Yang F, Lau T, Luo N. Cost-effectiveness of haemodialysis and peritoneal dialysis for patients with end-stage renal disease in Singapore. Nephrol Carlton Vic 2016;21(8):669–677; doi: 10.1111/nep.12668 26566750

[49] Ministry of Health (MOH). MOH | Fee Benchmarks and Bill Amount Information. n.d. Available from: https://www.moh.gov.sg/cost-financing/fee-benchmarks-and-bill-amount-information [Last accessed: May 2, 2023].

[50] Ministry of Manpower (MOM). Singapore Yearbook of Manpower Statistics 2022: Income, Wages, Earnings and Labour Cost Table(s). n.d. Available from: https://stats.mom.gov.sg/Pages/Singapore-Yearbook-Of-Manpower-Statistics-2022-Income-Wages-Earnings-and-Labour-Cost.aspx [Last accessed: May 2, 2023].

[51] OANDA. Currency Converter | Foreign Exchange Rates | OANDA. n.d. Available from: https://www.oanda.com/currency-converter/en/ [Last accessed: November 15, 2023].

[52] Petrovski G, Al Khalaf F, Campbell J, et al. Glycemic outcomes of Advanced Hybrid Closed Loop system in children and adolescents with type 1 diabetes, previously treated with Multiple Daily Injections (MiniMed 780G system in T1D individuals, previously treated with MDI). BMC Endocr Disord 2022;22(1):80; doi: 10.1186/s12902-022-00996-7 35351095 PMC8962027

[53] Department of Statistics, Singapore. National Accounts—Latest Data. n.d. Available from: https://www.singstat.gov.sg/find-data/search-by-theme/economy/national-accounts/latest-data [Last accessed: May 2, 2023].

[54] Zhao YJ, Khoo AL, Lin L, et al. Cost-effectiveness analysis of ticagrelor and prasugrel for the treatment of acute coronary syndrome. Value Health Reg Issues 2016;9:22–27; doi: 10.1016/j.vhri.2015.07.001 27881255

[55] Chua WBB, Cheen HHM, Kong MC, et al. Modelling the cost-effectiveness of pharmacist-managed anticoagulation service for older adults with atrial fibrillation in Singapore. Int J Clin Pharm 2016;38(5):1230–1240; doi: 10.1007/s11096-016-0357-7 27461367

[56] Wang Y, Xie F, Kong MC, et al. Cost-effectiveness of dabigatran and rivaroxaban compared with warfarin for stroke prevention in patients with atrial fibrillation. Cardiovasc Drugs Ther 2014;28(6):575–585; doi: 10.1007/s10557-014-6558-1 25319314

[57] Tornese G, Buzzurro F, Carletti C, et al. Six-month effectiveness of advanced vs. Standard Hybrid Closed-Loop System in children and adolescents with type 1 diabetes mellitus. Front Endocrinol 2021;12:766314; doi: 10.3389/fendo.2021.766314 PMC8630740 34858339

[58] Agency for Care Effectiveness (ACE). Opportunities for Patient Involvement. n.d. Available from: https://www.ace-hta.gov.sg/Patients-And-Community/opportunities-for-patient-involvement [Last accessed: May 2, 2023].

[59] Ministry of Health (MOH). MOH | Healthcare Schemes & Subsidies. n.d. Available from: https://www.moh.gov.sg/healthcare-schemes-subsidies [Last accessed: May 3, 2023].

[60] Medtronic. Decision Makers in Europe Further Expand Patient Access for Technology That Simplifies Diabetes Management. n.d. Available from: https://news.medtronic.com/Decision-makers-in-Europe-further-expand-patient-access-for-technology-that-simplifies-diabetes-management [Last accessed: August 1, 2023].

[61] The National Institute for Health and Care Excellence (NICE). Hybrid Closed Loop Systems for Managing Blood Glucose Levels in Type 1 Diabetes. n.d. Available from: https://www.nice.org.uk/guidance/indevelopment/gid-ta10845 40245209

[62] Si L, Willis MS, Asseburg C, et al. Evaluating the ability of economic models of diabetes to simulate new cardiovascular outcomes trials: A report on the Ninth Mount Hood Diabetes Challenge. Value Health 2020;23(9):1163–1170; doi: 10.1016/j.jval.2020.04.1832 32940234

[63] Tew M, Willis M, Asseburg C, et al. Exploring structural uncertainty and impact of health state utility values on lifetime outcomes in diabetes economic simulation models: Findings from the Ninth Mount Hood Diabetes Quality-of-Life Challenge. Med Decis Mak 2022;42(5):599–611; doi: 10.1177/0272989X211065479 PMC9329757 34911405

[64] van Duinkerken E, Snoek FJ, de Wit M. The cognitive and psychological effects of living with type 1 diabetes: A narrative review. Diabet Med 2020;37(4):555–563; doi: 10.1111/dme.14216 31850538 PMC7154747

[65] Ng SM, Corbett T, Doble E, et al. Managing the psychosocial impact of type 1 diabetes in young people. BMJ 2022;377:e070530; doi: 10.1136/bmj-2022-070530 35379655

[66] Chatterjee S, Bakhla AK, Biswas P, et al. Psychosocial morbidity among children with type-1 diabetes mellitus. J Fam Med Prim Care 2020;9(2):652; doi: 10.4103/jfmpc.jfmpc_1216_19 PMC7114057 32318398

[67] Emral R, Pathan F, Cortés CAY, et al. Self-reported hypoglycemia in insulin-treated patients with diabetes: Results from an international survey on 7289 patients from nine countries. Diabetes Res Clin Pract 2017;134:17–28; doi: 10.1016/j.diabres.2017.07.031 28951336

[68] The National Institute for Health and Care Excellence (NICE). Overview | Type 1 Diabetes in Adults: Diagnosis and Management. 2022. Available from: https://www.nice.org.uk/guidance/ng17 32017485

