# Supplementary material for: The Cost-Effectiveness of an Advanced Hybrid Closed-Loop System Compared to Standard Management of Type 1 Diabetes in a Singapore Setting
Source: Diabetes Technol Ther. 2024 Apr 30;26(5):324–34. doi: 10.1089/dia.2023.0455 (PMC11058413; doi:10.1089/dia.2023.0455)
Supplement: Supplemental data [file Suppl_TableS1.pdf]

Supplementary Table S1. List of currencies with conversion factors

Currency	Conversion Factor (1 SGD to Currency) As of November 2023[52]
USD (United States Dollar)	0.735
EUR (Euro)	0.626
AUD (Australian Dollar)	1.33
GBP (British Pound Sterling)	0.59
